# Total Cerebral Small Vessel Score Association With Hoehn and Yahr Stage in Parkinson’s Disease

**DOI:** 10.3389/fnagi.2021.682776

**Published:** 2021-05-28

**Authors:** Xinxin Ma, Shuhua Li, Chunmei Li, Rui Wang, Min Chen, Haibo Chen, Wen Su

**Affiliations:** ^1^Department of Neurology, Parkinson’s Disease and Extra Pyramidal Disease Diagnosis and Treatment Center, Beijing Hospital, National Center of Gerontology, Institute of Geriatric Medicine, Chinese Academy of Medical Sciences, Beijing, China; ^2^Department of Radiology, Beijing Hospital, National Center of Gerontology, Institute of Geriatric Medicine, Chinese Academy of Medical Sciences, Beijing, China

**Keywords:** Parkinson’s disease, cerebral small vessel disease, lacunes, white matter hyperintensities, enlarged perivascular spaces, cerebral microbleeds

## Abstract

**Background**: This study aimed to evaluate the total cerebral small vessel disease (CSVD) score in patients with Parkinson’s disease (PD) at different stages and related factors.

**Methods**: A 100 and seven patients with idiopathic PD and 62 normal controls (NCs) who underwent brain magnetic resonance imaging (MRI) were enrolled. PD patients were divided into two groups: early PD [(Hoehn and Yahr (H&Y) 1–1.5, *n* = 36)] and advanced PD (H&Y 2–4, *n* = 71) groups. We calculated the total CSVD score for each participant based on lacunes, high-grade white matter hyperintensities (WMH), enlarged perivascular spaces (EPVS), and cerebral microbleeds (CMBs). Differences in total CSVD score between the PD and NCs and between the two subgroups were compared. In addition, a multivariate logistic regression analysis was conducted to investigate the association between CSVD markers and clinical variables in PD.

**Results**: Lacunes were found in 9.3% of patients with PD, periventricular WMH (PVWMH) in 89.7%, deep WMH (DWMH) in 81.3%, EPVS in 85%, and CMBs in 2.8%. Compared with NCs, patients with PD showed higher PVWMH and DWMH scores. Advanced PD patients exhibited greater PVWMH (*P* = 0.041), DWMH (*P* = 0.046), and total CSVD score (*P* = 0.044) than the early PD group. After adjusting for multiple variables, higher H&Y stage was independently correlated with increased total CSVD score (OR = 2.667, 95% CI 1.154–2.266) and PVWMH score (OR = 2.237, 95% CI 1.084–1.696).

**Conclusions**: CSVD may play a critical role in patients with PD. The total CSVD score is a potential neuroimaging marker for monitoring the progression of PD.

## Introduction

Parkinson’s disease (PD) is a common neurodegenerative disease, with so far an unclear underlying mechanism. The contribution of vascular pathology to PD is receiving increasing attention. However, there were controversial reports on the relationship between vascular disease and PD. Cerebral small vessel disease (CSVD) comprises a group of disorders of various etiologies that affect the small arteries, arterioles, venules, and capillaries in the brain (Pantoni, 2010). On brain MRI, CSVD can present as lacunes, white matter hyperintensities (WMH), enlarged perivascular spaces (EPVS), and cerebral microbleeds (CMBs; Pantoni, 2010). The total CSVD score has been used to assess neuroimaging markers in CSVD, including lacunes, high-grade WMH, EPVS in the basal ganglia, and CMBs, which might be better than separately measuring only one or two features (Klarenbeek et al., 2013; Staals et al., 2014). The total CSVD score, is, therefore, a more complete estimate of the full impact of CSVD on the brain (Staals et al., 2014).

CSVD has been shown to contribute to motor and cognitive functions in PD (Linortner et al., 2020). Previous work has also demonstrated that WMH is correlated with motor dysfunction and several non-motor symptoms in PD (Lee et al., 2018, 2020; Huang et al., 2020). However, other CSVD markers have received little attention. Only three studies calculated the total CSVD burden in patients with PD. Their findings indicated that CSVD burden was related to motor symptoms (especially gait/postural instability), cognitive impairment, and affective disorders (Shibata et al., 2019; Chen et al., 2020, 2021). Another autopsy study also revealed the severity of SVD pathology characterized by globus pallidus interna pallor associated with Hoehn and Yahr (H&Y) stage. However, the interaction effect between CSVD burden and H&Y stage in PD has not yet been reported. It is still unclear whether comorbid CSVD exacerbates the progression of PD.

The H&Y stage is a widely used scale for evaluating disease progression in PD (Hoehn and Yahr, 1967; Goetz et al., 2004), while the H&Y transition time is also considered a useful measure of disease progression in PD (Zhao et al., 2010). Several neuroimaging studies have shown that the H&Y stage correlates with progressive nigrostriatal terminal dysfunction (Vingerhoets et al., 1994; Staffen et al., 2000). These findings support the usefulness of the H&Y stage for categorizing patients with PD and capturing disease progression.

In our study, we aimed to investigate the total CSVD burden in patients with PD at different stages based on the H&Y scale. We also examined factors related to the total CSVD score and other CSVD markers in PD. This study may help elucidate the relationship between CSVD and PD and identify potential neuroimaging markers for diagnosing and monitoring PD progression.

## Materials and Methods

### Subjects and Clinical Assessments

Patients with idiopathic PD (*n* = 107, mean age: 66.20 ± 8.69 years) and age-and sex-matched normal controls (NCs; *n* = 62, mean age: 65.69 ± 6.45 years) were recruited. All PD patients were diagnosed based on the UK Parkinson’s Disease Society Brain Bank Clinical Diagnostic Criteria. All participants were right-handed Chinese natives. We excluded patients whose PD was induced by cerebrovascular disease, medications, trauma, encephalitis, poisoning, and other neurodegenerative diseases. Vascular risk factors were recorded, including hypertension, diabetes mellitus, hyperlipidemia, coronary heart disease, atrial fibrillation, and smoking status. Neurological examinations were evaluated using the Mini-Mental State Examination (MMSE), Unified Parkinson’s Disease Rating Scale part III score (UPDRS-III), H&Y Stage, Hamilton Rating Scale for Depression (HAMD), Hamilton Rating Scale for Anxiety (HAMA), Parkinson’s disease questionnaire-39 (PDQ-39), and non-motor symptoms questionnaire (NMSQ). Patients with PD receiving dopaminergic medications were examined in a clinically defined “OFF” state. All neuropsychological scales were completed by a neurologist blinded to clinical diagnosis. Patients with obvious cognitive deficits were excluded (MMSE score ≤24). Patients were classified into the early (H&Y 1–1.5) and advanced PD groups (H&Y 2–4), based on the H&Y stage. This study was approved by a local ethics committee, and written informed consent was obtained from each participant after a detailed description of the study was provided.

### MR Image Acquisition

All MRI examinations were performed using a 3.0 T MRI scanner (Philips, Achieva TX, 8-channel high-resolution head coil). Sequences consisted of high-resolution T1-weighted 3D [repetition time/echo time (TR/TE) = 7.4/3 ms, flip angle (FA) = 8°, field of view (FOV) = 24 cm × 24 cm, matrix = 256 × 256, and 1.2 mm slice thickness without slice gap], T2-weighted (T2WI, TR/TE = 2,500/100 ms; FOV = 24 cm × 24 cm, matrix = 256 × 256, 5 mm slice thickness, and 1.5 mm slice gap), fluid-attenuated inversion recovery (FLAIR; TR/TE = 8,000/140 ms; TI = 2,400 ms; FOV = 24 cm × 24 cm, matrix = 256 × 228, and 4 mm slice thickness without slice gap), diffusion-weighted imaging (DWI; TR/TE = 5,000/76.4 ms; matrix = 128 × 128, and 5 mm slice thickness), susceptibility-weighted imaging (SWI; TR/TE = 16/22 ms; FOV = 24 cm × 24 cm, matrix = 240 × 240, and 2.8 mm slice thickness without slice gap).

### MRI Analysis

**Figure 1:**
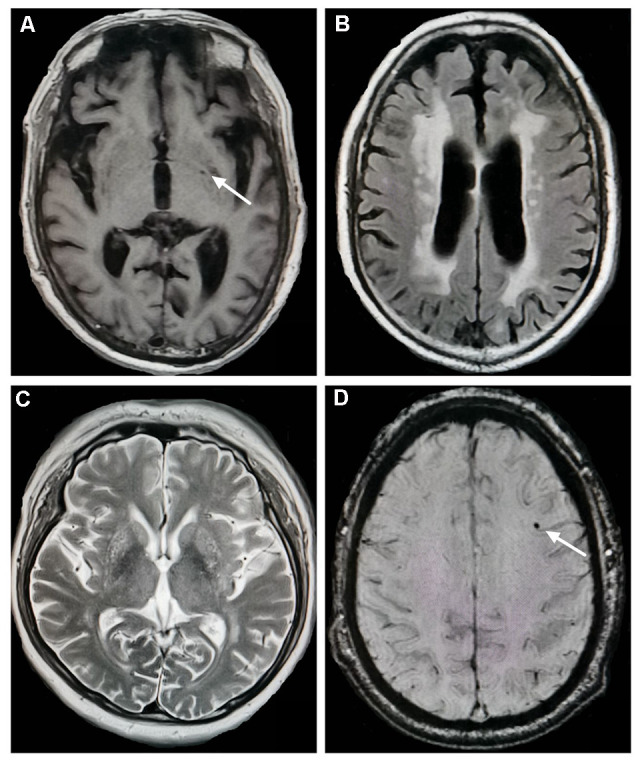
Neuroimaging markers of cerebral small vessel disease. **(A)** Lacunes (white arrow) in the left basal ganglia (T1-weighted imaging). **(B)** Severe periventricular white matter hyperintensities (WMH; fluid-attenuated inversion recovery). **(C)** Enlarged perivascular spaces in the basal ganglia (T2-weighted imaging). **(D)** Cerebral microbleeds (white arrow) in the left frontal lobe (susceptibility-weighted imaging).

CSVD markers include lacunes, WMH, EPVS, and CMBs (Figure 1). Lacunes were defined as round or ovoid cerebrospinal fluid-filled cavities in the basal ganglia or white matter, usually 3–15 mm, with low signal on T1WI and DWI, and high signal on T2WI (Wardlaw, 2008; Wardlaw et al., 2013). Periventricular WMH (PVWMH) and deep WMH (DWMH) lesions were investigated using the Fazekas scale from 0 to 3 (Fazekas et al., 1987). PVWMH was defined as 0 = absence, 1 = “caps” or pencil-thin lining, 2 = smooth “halo” and 3 = irregular PVWMH extending into the deep white matter. DWMH refers to 0 = absence, 1 = punctate foci, 2 = beginning confluence of foci, 3 = large confluent areas)Fazekas et al., 1987). EPVS were defined as punctate hyperintensities on T2WI in the basal ganglia, usually <3 mm in diameter, based on a previous study (Doubal et al., 2010). Isolated single large invaginations of cerebrospinal fluid round perforating vessels were not counted. EPVS were rated as follows: 0 = no EPVS, 1 = <10 EPVS, 2 = 11–20 EPVS, 3 = 21–40 EPVS, and 4 = >40 EPVS. If there was an asymmetry between the sides, the hemisphere most affected was calculated (Doubal et al., 2010). CMBs are well-defined, round hypointensities, ≤10 mm on SWI images (Wardlaw et al., 2013). All MRI lesions were assessed by two trained neurologists blinded to the participants’ clinical information.

### Total CSVD Burden/Score

Based on the description by Staals et al., we calculated the total CSVD score on an ordinal scale from 0 to 4. One point on the CSVD score was awarded for each of the following: ≥1 lacunes, ≥1 CMBs, high-grade WMH (Fazekas score = 3 in PVWMH or ≥2 in DWMH), and moderate-to-severe EPVS (>10 in the basal ganglia; Staals et al., 2014).

### Statistical Analysis

The Statistical Package for the Social Sciences (SPSS) software (version 19.0) was used to analyze clinical and demographic variables. A two-sample *t*-test and Chi-square test were conducted to examine the clinical differences between continuous and categorical variables, respectively. The Mann–Whitney *U* test was used to compare the differences in H&Y stage and CSVD markers between groups. Multivariate ordered logistic regression analysis was performed between the total CSVD score and the clinical variables in PD. The total CSVD score was the dependent variable, and clinical factors were independent variables, including age, sex, vascular risk factors, years of education, disease duration, LED, UPDRS-III, H&Y stage, MMSE, HAMD, and HAMA score. Factors related to each CSVD marker (lacunes, WMH, EPVS, and CMBs) were further analyzed. Statistically significant was set at *p* < 0.05.

## Results

In this study, we recruited 107 patients with PD and 62 NCs. Demographic and clinical data are presented in Table 1. NCs had a higher proportion of diabetes mellitus, hypercholesterolemia, coronary heart disease, and current smoking. The two groups did not differ in terms of age, sex, prevalence of hypertension, and atrial fibrillation. In the PD group, lacunes were present in 9.3%, PVWMH in 89.7%, DWMH in 81.3%, EPVS in 85%, and CMBs in 2.8% patients. Compared with NCs, PD patients showed higher PVWMH (*U* = 2,720.50, *P* = 0.039) and DWMH scores (*U* = 2,658.50, *P* = 0.011). However, there were no significant differences in lacunes, BG-EPVS, CMBs, and total CSVD score between patients with PD and controls.

**Table 1:** Demographic and clinical data of the subjects.

	PD (*n* = 107)	NCs (*n* = 62)	*t*/*x*^2^	*P*
Age (year)	66.20 ± 8.69	65.69 ± 6.45	−0.429	0.669
Sex (M/F)	56/51	29/33	0.486	0.486
Hypertension (%)	35 (32.7)	28 (45.2)	2.603	0.107
Diabetes mellitus (%)	12 (11.2)	23 (37.1)	18.193	0.000*
Hyperlipidemia (%)	32 (29.9)	32 (51.6)	7.861	0.005*
Coronary heart	17 (15.9)	19 (30.6)	5.099	0.024*
disease (%)
Atrial fibrillation (%)	1 (0.9)	0 (0.0)	0.583	0.445
Current smoking (%)	3 (2.8)	7 (11.3)	5.078	0.024*

*Chi-square tests, *P* < 0.05.

**Figure 2:**
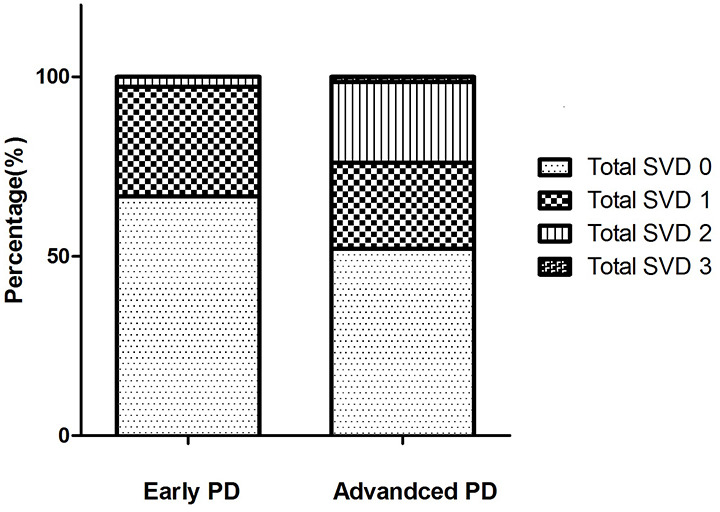
The percentage of different total cerebral small vessel disease (CSVD) scores in the two Parkinson’s disease (PD) subgroups. In early PD group, the total CSVD score 0–3 were present in 66.7%, 30.6%, 2.8%, 0% of the patients, respectively. In advanced PD group, the total CSVD score 0–3 were present in 52.1%, 23.9%, 22.5%, 1.4% of the patients, respectively. There were no patients with a score of 4 in our study.

Compared with the early PD group, advanced PD patients showed higher levodopa equivalent dose (LED), higher UPDRS-III, and UPDRS total score, longer disease duration, higher PDQ-39 score, and lower smoking rate (Table 2). The two groups did not differ in age, sex ratio, education years, and vascular risk factors except smoking status, MMSE, HAMD, HAMA, and NMSQ scores. Figure 2 shows the percentages of different CSVD scores in the two subgroups. Patients with advanced PD exhibited greater PVWMH, DWMH, and total CSVD scores than the early PD group (Table 2). After adjusting for smoking status, advanced PD patients still showed greater PVWMH (*F* = 4.935, *P* = 0.028), DWMH (*F* = 5.824, *P* = 0.018), and total CSVD score (*F* = 5.121, *P* = 0.026) than the early PD group. However, there was no significant difference in lacunes, EPVS, and CMBs between the two subgroups.

**Table 2:** Demographic and total CSVD score in the early and advanced PD groups.

	ePD (*n* =36)	aPD (*n* =71)	*t*/*x*^2^/*U*	*P*
Age (year)	66.36 ± 9.26	66.11 ± 8.45	0.139	0.890
Sex (M/F)	19/17	37/34	0.004	0.948
Hypertension (%)	13 (36.1)	22 (31.0)	0.285	0.593
Diabetes mellitus (%)	5 (13.9)	7 (9.8)	0.390	0.533
Hypercholesterolemia (%)	7 (19.4)	25 (35.2)	2.833	0.092
Coronary heart disease (%)	5 (13.9)	12 (16.9)	0.162	0.687
Atrial fibrillation (%)	0 (0)	1 (1.4)	0.512	0.474
Current smoking (%)	3 (8.3)	0 (0)	6.087	0.036^b
LED (mg/day)	287.85 ± 247.81	474.36 ± 305.12	−3.173	0.002^a
UPDRS-III	15.97 ± 6.29	31.63 ± 11.74	−8.980	0.000^a
UPDRS total	23.44 ± 8.00	37.34 ± 13.31	−6.722	0.000^a
H&Y stage	1.2 (1-1.5)	2.5 (2-4)	−17.183	0.000^a
Education	13.67 ± 3.36	13.21 ± 2.78	0.746	0.457
Duration	5.11 ± 4.35	8.48 ± 4.39	−3.761	0.000^a
MMSE	28.69 ± 1.60	28.13 ± 1.63	1.713	0.090
HAMD	7.31 ± 4.74	9.01 ± 5.21	−1.650	0.102
HAMA	9.00 ± 5.53	9.92 ± 5.64	−0.798	0.426
PDQ-39	16.61 ± 12.31	28.97 ± 19.31	−4.019	0.000^a
NMSQ	10.42 ± 3.77	11.83 ± 4.86	−1.528	0.130
MRI features				
Lacunes (%)	1 (2.8)	9 (12.7)	1.718	0.190
PVWMH (IQR)	1.33 (0–3)	1.70 (0–3)		0.041^c
DWMH (IQR)	0.81 (0–2)	1.07 (0–3)	1,025.5	0.046^c
EPVS (IQR)	1.03 (0–3)	1.32 (0–4)	1,082	0.145
CMBs (%)	2 (5.6)	1 (1.4)	1.508	0.261
CSVD burden (IQR)	0.36 (0–2)	0.73 (0–3)	1,006.5	0.044^c

*ePD, early PD; aPD, advanced PD; LED, levodopa equivalent dose; MMSE, Mini-Mental State Examination, UPDRS, Unified Parkinson’s Disease Rating Scale; H&Y stage, Hoehn and Yahr stage; HAMD, Hamilton Rating Scale for Depression; HAMA, Hamilton Rating Scale for Anxiety; PDQ-39, the Parkinson’s disease questionnaire-39; NMSQ, non-motor symptoms questionnaire; IQR, interquartile range; ^a^Independent *t*-test, *P* < 0.05. ^b^Chi-square test, *P* < 0.05. ^c^Mann-Whitney *U* test, *P* < 0.05*.

In multivariate ordered logistic regression analysis, the higher H&Y stage was independently correlated with increased total CSVD (OR = 2.667, 95% CI 1.154–2.266, *P* = 0.022) and PVWMH score (OR = 2.237, 95% CI 1.084–1.696, *P* = 0.029; Table 3). However, there was no significant association between the total CSVD score and sex, years of education, disease duration, LED, UPDRS-III, MMSE, HAMD, HAMA, PDQ-39, NMSQ, and vascular risk factors such as hypertension, hyperlipidemia, smoking, and cardiovascular disease. No marked relationships were demonstrated between the H&Y stage and lacunes, DWMH, EPVS, and CMBs scores. In addition, age was positively associated with PVWMH, DWMH, EPVS, and total CSVD scores. Diabetes mellitus was also related to increased DWMH, EPVS, and total CSVD burden (Table 3).

**Table 3:** Correlation analysis between the CSVD markers clinical variables in PD.

	Clinical variables	OR	95% CI	*X*^2^	*P*
Related to CSVD burden	H&Y stage	2.667	1.154–2.266	5.270	0.022*
	Age	1.305	1.186–1.435	30.098	0.000*
	DM	5.618	1.406–22.421	5.970	0.015*
Related to PVWMH	H&Y stage	2.237	1.084–1.696	4.753	0.029*
	Age	1.202	1.130–1.279	34.205	0.000*
Related to DWMH	Age	1.121	1.052–1.194	12.483	0.000*
	DM	4.609	1.098–19.317	4.363	0.037*
Related to EPVS	Age	1.158	1.090–1.232	21.943	0.000*
	DM	4.716	1.283–17.322	5.456	0.020*

*Multivariate ordered logistic regression analysis; DM, diabetes mellitus.

## Discussion

The present study suggested a significant difference in total CSVD score between the early and advanced PD groups. The H&Y stage was independently correlated with the total CSVD score, and as such a potential marker for monitoring PD progression.

In recent years, only three studies have investigated the association between CSVD and PD using the total CSVD score. Shibata et al. (2019) suggested a relationship between cognitive decline and increased CSVD score. While another two studies by indicated that comorbid CSVD may play a critical role in several PD domains, including motor deficits, cognition, depression, and anxiety (Chen et al., 2020, 2021). Although PD patients with more severe CSVD burden showed a higher H&Y stage, there were no significant differences in the H&Y stage between five subgroups according to the CSVD burden score. The association between CSVD burden and the H&Y stage had not been previously reported. In a cohort of 77 autopsy-confirmed PD patients, Schwartz et al. (2018) revealed the severity of SVD pathology characterized by globus pallidus interna pallor associated with the Hoehn and Yahr (H&Y) stage. Our study is the first to demonstrate a correlation between the total CSVD score and the H&Y stage in PD. The assessment of CSVD could be used as a clinically relevant neuroimaging marker in studies of disease progression in PD.

The pathologies of cerebrovascular diseases in PD have been investigated in several studies. The prevalence of cerebrovascular lesions in PD (44.0%) was higher than in controls (32.8%), including lacunes, amyloid angiopathy, white matter lesions, old and recent ischemic infarcts, and hemorrhages (Jellinger, 2003). However, another study reported opposite results (Schwartz et al., 2012). The vascular pathology of PD includes capillary fragmentation and damage to the capillary network in multiple brain regions, particularly in the substantia nigra, middle frontal cortex, and brain stem nuclei (Guan et al., 2013). Further, widespread cerebral blood flow reduction has also been observed in patients with PD (Fernandez-Seara et al., 2012). These findings suggest shared pathogenic pathways between cerebrovascular diseases and PD (Kummer et al., 2019). We speculate that comorbid CSVD may lead to more widespread disruption, which could exacerbate PD progression.

In addition to the total CSVD burden, WMH has been related to motor symptoms in PD, especially bradykinesia and axial symptoms (Bohnen et al., 2011; Lee et al., 2020; Jeong et al., 2021). Other studies also indicated that WMH correlated with motor subtype and gait in patients with PD (Bohnen et al., 2011; Al-Bachari et al., 2017; Toda et al., 2019; Wan et al., 2019). Furthermore, there is an association between WMH and several non-motor symptoms in PD, such as cognitive dysfunction, depression, anxiety, fatigue, and quality of life (Lee et al., 2018; Huang et al., 2020). In another longitudinal study, Pozorski et al. found that greater WMH accumulation correlated with increased UPDRS motor sub-scores and impaired cognitive performance over an 18-month period in PD patients (Pozorski et al., 2019). Their findings suggest that WMH may worsen motor and cognitive functions in patients with PD. Our results showed that PD patients had higher PVWMH scores than NCs, which was also independently associated with the H&Y stage. The present finding is in line with previous studies, suggesting that PVWMH may be a promising marker for diagnosing and monitoring PD disease progression. The mechanism underlying WMH was associated with vascular changes including arteriolar tortuosity, decreased vessel density, occlusive venous collagenosis, and reduced myelin density due to Wallerian degeneration secondary to neuron loss, and low-grade inflammation (Smith, 2010; Wersching et al., 2010; Bohnen and Albin, 2011). WMH could also disrupt connectivity in widespread neural systems and exacerbate some motor and cognitive deficits in PD (Bohnen and Albin, 2011). Hence, comorbid white matter disease may provide a new sight for PD.

Regarding other CSVD markers, previous work showed that lacunes in the basal ganglia independently correlated with impaired gait and posture dysfunction in patients with PD (Chen et al., 2020). Moreover, EPVS in the basal ganglia is related to the tremor score (Wan et al., 2019), and may be a predictor of cognitive impairment in PD (Shibata et al., 2019). Yamashiro et al. also revealed that deep or infratentorial CMBs were more frequent in PD; risk factors include hypertension, orthostatic hypotension, and a history of ischemic stroke (Yamashiro et al., 2015). Patients with the postural instability gait disorder (PIGD) subtype exhibited a higher prevalence of CMBs compared to NCs (Kim et al., 2018). PD patients with CMBs were older and had higher CSVD scores than those without (Kim et al., 2018). In a Chinese cohort study, a history of cerebral ischemic events and hypertension was independently associated with CMBs presence in PD (He et al., 2017). However, our results did not show any differences in lacunes, EPVS, and CMBs between PD patients and controls. No marked relationship between these CSVD markers and clinical variables was observed. We speculate that WMH may play a more critical role in PD than other CSVD markers. To prove this point, larger sample size studies in this field are needed in the future.

This study has several limitations: (1) The sample size was relatively small, therefore, future longitudinal studies are warranted. (2) We did not explore the relationship between UPDRS subscores and CSVD markers. (3) The total CSVD score is a semi-quantitative method, and future integrated studies using multimodal structural, functional, and metabolic neuroimaging techniques are needed to provide new insights into the interaction between CSVD and PD.

In conclusion, we found that total CSVD and WMH scores were independently associated with disease stage in PD. These scores may be promising markers for monitoring PD progression. Comorbid CSVD may be an aggravating factor for the progression of PD, and immediate clinical and public health implications. Therefore, screening for CSVD should be considered in PD. The management of vascular risk factors may be helpful in patients with PD.

## Data Availability Statement

The original contributions presented in the study are included in the article, further inquiries can be directed to the corresponding author.

## Ethics Statement

The studies involving human participants were reviewed and approved by Beijing Hospital Ethics Committee. The patients/participants provided their written informed consent to participate in this study.

## Author Contributions

XM, WS, and HC contributed to the conception and design of the study. SL, CL, RW, and MC organized the database. XM performed the statistical analysis, and wrote the first draft of the manuscript. All authors contributed to the article and approved the submitted version.

## Conflict of Interest

The authors declare that the research was conducted in the absence of any commercial or financial relationships that could be construed as a potential conflict of interest.
